# Can red cell distribution width in very low birth weight infants predict bronchopulmonary dysplasia?

**DOI:** 10.1097/MD.0000000000028640

**Published:** 2022-01-21

**Authors:** Seong Hee Oh, Hyun-Jeong Do, Ji Sook Park, Jae Young Cho, Chan-Hoo Park

**Affiliations:** a Department of Pediatrics, University of Ulsan College of Medicine, Gangneung Asan Hospital, Gangneung, South Korea; b Department of Pediatrics, College of Medicine, Gyeongsang National University, Gyeongsang National University Hospital, Jinju, South Korea; c Institute of Health Sciences, Gyeongsang National University, Jinju, South Korea; d Department of Pediatrics, College of Medicine, Gyeongsang National University, Gyeongsang National University Changwon Hospital, Changwon, South Korea.

**Keywords:** bronchopulmonary, dysplasia, infant, premature, red cell distribution width, very low birth weight infant

## Abstract

Red cell distribution width (RDW) is a useful marker for assessing the severity and prognosis of various diseases in adults. However, whether it is applicable to children, especially in newborns, has not been determined.

This study aimed to investigate the RDW values of preterm infants and evaluate whether RDW values in the early days of life can predict bronchopulmonary dysplasia (BPD) development.

One hundred and eight infants born at <30 weeks of gestation with a birth weight of <1500 g participated in this retrospective study. RDW values measured at birth, 7 days (D7), and 28 days (D28) after birth were reviewed. The changes in RDW values in the first month of life were analyzed, and we evaluated the relationship between RDW and BPD.

The mean RDW values at birth, D7, D28 and the change from birth to D7 were 16.2 ± 0.1%, 17.5 ± 0.2%, 17.6 ± 0.2% and 1.3 ± 1.8%, respectively. RDW at birth was lower in the infants born at <28 weeks’ gestational age than in those born at ≥28 weeks’ gestational age (15.7 ± 0.3 vs 16.4 ± 0.2, *P* = .024). RDW values of both groups increased during the first week after birth and did not differ significantly at D7. The levels remained similar at 1 month of age. RDW at birth, D7, and D28 and the changes in RDW from birth to D7 were not correlated with the development of BPD independent of its severity.

The usefulness of RDW as a predictor of BPD development remains questionable and requires further study.

## 1 Introduction

Red cell distribution width (RDW) as part of complete blood count (CBC) has traditionally been used with mean corpuscular volume (MCV) to determine the cause of anemia. RDW values increase in circumstances of ineffective erythropoiesis in the bone marrow such as iron deficiency anemia, folate and vitamin B12 deficiency, or shortening of the red blood cell (RBC) life span by destruction such as in sickle cell anemia.^[1]^

Many recent studies have reported that RDW is related to inflammation^[2–4]^ and hypoxemia,^[5]^ and is an easily available parameter that can predict the severity^[4,6,7]^ and prognosis of various diseases in adults.^[3,7–17]^ Additional blood sampling is not required to determine the RDW values, as CBC is performed relatively frequently and requires only a small amount of blood. Therefore, RDW might be a useful tool for assessing the medical conditions of newborns, especially preterm infants. However, there are no sufficient studies in children and infants on this topic.^[18–23]^ Moreover, there are few studies on preterm infants, and the normal range of RDW values in preterm infants has yet to be determined.^[24–26]^

Bronchopulmonary dysplasia (BPD), which is a major complication of preterm infants, occurs when premature lung tissue and vessels are injured and their development and differentiation are disrupted by perinatal inflammation, such as chorioamnionitis, hyperoxia, mechanical ventilation, and infection. There have been a few previous studies on the relationship between RDW and BPD, but they have shown conflicting results.^[25–27]^ The authors investigated the changes in RDW values in very low birth weight infants born before 30 weeks of gestation and whether the RDW values measured in the early days of life can predict BPD development.

## 2 Methods

This study was a retrospective review of the medical records of patients hospitalized in the two-level lll neonatal intensive care units at Gyeongsang National University Hospital and Changwon Gyeongsang National University Hospital in South Korea between January 2009 and August 2019. This study was approved by the Institutional Review Boards of the Gyeongsang National University Hospital (IRB no. 2020-02-014) and Changwon Gyeongsang National University Hospital (IRB no. 2020-02-017), which waived the need for informed consent. All methods were performed in accordance with the relevant guidelines and regulations.

Patients born at gestational age (GA) <30 weeks and birth weight <1500 g were included in this study. Patients with

1. a chromosomal abnormality or major congenital anomaly;
2. culture-proven early-onset sepsis;
3. a recent RBC transfusion (within 1 week after birth); or
4. maternal anemia (hemoglobin <8 g/dL) were excluded.

RDW values measured at birth, 7 days (D7), and 28 days (D28) after birth were reviewed. The RDW levels according to GA and the association between RDW and BPD development were analyzed. RDW was determined as part of a CBC, which was checked within 1 hour after birth, at D7, D28, and per the local routine protocol as necessary. The 0.3 to 0.5 mL of Blood samples were taken from the radial or umbilical artery and placed in ethylenediaminetetraacetic acid-containing tubes. CBC was measured using an automated hematology analyzer (Sysmex, Kobe, Japan) and quality controls and calibrations were performed regularly according to standard rules. White blood cell counts, hemoglobin, MCV, and platelet counts were also collected using the RDW from the CBC.

BPD was defined according to the National Institute of Child Health and Human Development consensus definition.^[28]^ BPD was diagnosed if the patient required artificial respiratory support providing positive airway pressure or oxygen for more than 28 days. Moderate or severe BPD was diagnosed if the patient needed positive airway pressure or oxygen at 36 weeks’ postmenstrual age or at discharge. Babies who died in the first month of life were excluded from the assessment of the relationship between RDW values and BPD development.

Statistical analysis was performed using SAS software (version 9.4; SAS Institute, Inc., Cary, NC, USA). The normality of continuous values was assessed using the Kolmogorov-Smirnov test. Categorical and continuous variables are presented as number (percentage) and mean ± standard deviation or median (interquartile range [IQR]), respectively. Categorical variables were analyzed using the Chi-Squared test or Fisher exact test. Continuous variables were analyzed using the Student *t* test or Mann––Whitney's *U* test. A linear mixed-effects model was used to analyze the trends of RDW levels during the first month of life and compare them between infants born at <28 weeks’ GA and those born at ≥28 weeks’ GA. Multivariable logistic regression was used to analyze the association between RDW and BPD. Statistical significance was set at *P* < .05.

## 3 Results

### 3.1 Characteristics of the infants

Of the 171 infants eligible for this study, 63 were excluded. The mean GA and birth weight of the remaining 108 patients were 28.4 ± 1.4 weeks’ gestation and 1165.6 ± 212.9 g. The number of infants with BPD and moderate/severe BPD were 64 (59.3%) and 21 (19.4%), respectively. White blood cell count and C-reactive protein were higher in infants born at < 28 weeks’ GA (n = 27) than 28 to 29 weeks’ GA (n = 81). BPD and moderate/severe BPD presented more in the infants born before 28 weeks of gestation than in the infants born at 28 weeks of gestation or later (Table 1).

**Table 1:** Demographic characteristics and laboratory data by gestational age.

	GA < 28 weeks (n = 27)	GA 28–29 weeks (n = 81)	*P* value
Birth weight (g)	976.9 ± 213.2	1228.5 ± 172.8	<.001
SGA (n, %)	3 (11.1)	9 (11.1)	1.000
Male (n, %)	14 (51.9)	54 (66.7)	.167
C-sec (n, %)	16 (59.3)	62 (76.5)	.135
Maternal hypertension (n, %)	5 (18.5)	18 (22.2)	.684
WBC (10^3^/μL)	11.1 (5.9–15.9)	5.7 (4.1–8.2)	<.001
Hemoglobin (g/dL)	15.5 ± 1.0	16.0 ± 1.6	.059
MCV (fL)	115.6 ± 6.4	115.4 ± 6.0	.855
Platelet (10^3^/μL)	256.1 ± 69.1	232.2 ± 67.8	.118
CRP (mg/L)	0.3 (0.2–0.4)	0.2 (0.1–0.2)	.003
BPD (n, %)	21 (91.3)^∗^	43 (53.1)	<.001
Moderate BPD (n, %)	12 (52.2)^∗^	9 (11.1)	<.001

### 3.2 RDW values in preterm infants

**Figure 1:**
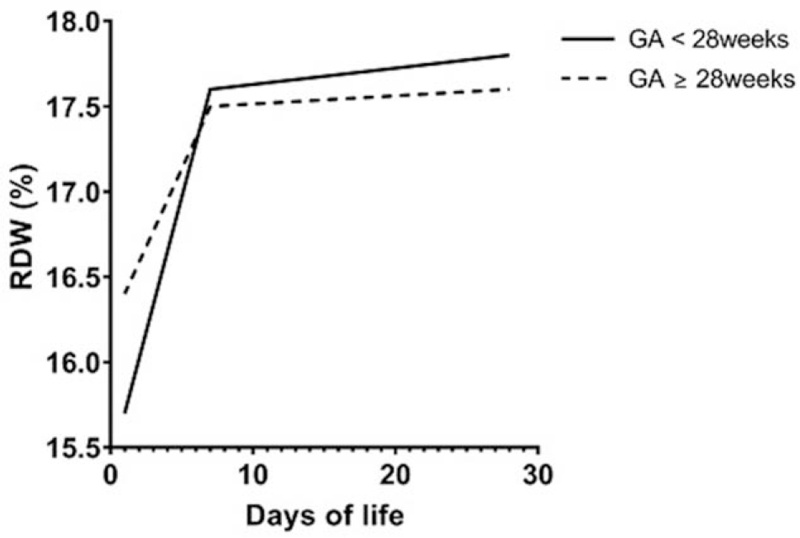
The trends of RDW in preterm infants born at <28 weeks’ GA versus those born at ≥28 weeks’ GA. The RDW increased during the first week after birth and remained similar for the first month independent of GA. GA = gestational age, RDW = red cell distribution width.

The mean RDW values at birth, D7, and D28 are presented in Table 2. The mean RDW value at birth was 16.2 ± 0.1%. The RDW increased during the first week after birth (*P* < .001) and did not change significantly from D7 to D28 (*P* = .658). The mean RDW value at birth was lower in infants born at <28 weeks’ GA than in those born at ≥28 weeks’ GA (*P* = .024). The RDW of both groups increased in the first week of life (*P* < .001, both) and then remained at similar levels for a month after birth (*P* = .639 and *P* = .772, respectively) without differences between the groups (Table 2, Fig. 1).

**Table 2:** Changes in RDW levels during the first month of life and the differences in RDW changes between infants born at <28 weeks’ GA and those born at ≥28 weeks’ GA.

	At birth	D7	D28	*P* value						
				Interaction effect	Time effect			Group effect		
					Overall	At birth vs D7	D7 vs D28	At birth	D7	D28
All	16.2 ± 0.1	17.5 ± 0.2	17.6 ± 0.2		<0.001	<0.001	0.658			
GA <28	15.7 ± 0.3	17.6 ± 0.5	17.8 ± 0.3	0.012	<0.001	<0.001	0.639	0.024	0.832	0.501
GA ≥28	16.4 ± 0.2	17.5 ± 0.3	17.6 ± 0.2		<0.001	<0.001	0.772			

### 3.3 Relationship between RDW and BPD

After 4 patients who died in the first month of life were excluded, the relationship between RDW and BPD development in the remaining 104 patients was evaluated. The RDW values at birth, D7, the change of RDW between birth and D7, and D28 did not show any difference in accordance to BPD and moderate/severe BPD. These results remained similar even after we divided the patients into 2 groups born <28 weeks of GA and 28 to 29 weeks of GA. (Table 3). In multivariable logistic regression, the RDW values were not related to BPD presentation regardless of BPD severity (Table 4).

**Table 3:** Comparison of RDW values between newborns with BPD and those without BPD in groups separated by gestational age.

	Total			GA < 28 weeks			GA 28–29 weeks		
	BPD (n = 64)	NonBPD (n = 40)	*P* value	BPD (n = 21)	NonBPD (n = 2)	*P* value	BPD (n = 43)	NonBPD (n = 38)	*P* value
RDW at birth	16.0 (15.5–16.8)	15.9 (15.3–16.7)	.390	15.8 (15.3–16.3)	15.9	0.783	16.3 (15.5–17.0))	15.9 (15.2–16.7)	.138
RDW at D7	17.3 (16.1–18.4)	16.8 (15.7–17.6)	.167	17.0 (16.0–18.9)	18.5	0.561	17.3 (16.2–18.3)	16.8 (15.6–17.6)	.105
RDW at D28	17.8 (16.7–18.9)	17.1 (16.2–18.7)	.282	17.9 (17.2–17.9)	17.4	0.640	17.8 (16.6–18.8)	17.1 (16.2–18.8)	.382
RDW birth-D7	0.9 (0.3–2.1)	0.8 (0.1–1.2)	.299	1.0 (0.3–2.7)	2.6	0.522	0.8 (0.3–1.8)	0.8 (0.1–1.1)	.349

	Moderate/severe BPD (n = 21)	Non- moderate/severe BPD (n = 83)	*P* value	Moderate/severe BPD (n = 12)	Nonmoderate/severe BPD (n = 11)	*P* value	Moderate/severe BPD (n = 9)	Non- moderate/severe BPD (n = 72)	P value
RDW at birth	15.8 (15.5–16.7)	16.2 (15.5–16.7)	0.650	15.8 (15.2–16.2)	15.8 (15.5–16.3)	0.797	15.8 (15.4–19.2))	16.2 (15.5–16.8)	0.964
RDW at D7	17.2 (16.1–20.0)	17.0 (16.1–18.2)	0.419	17.7 (16.3–21.2)	16.5 (15.9–18.7)	0.174	16.9 (15.7–20.3)	17.1 (16.1–18.3)	0.792
RDW at D28	17.9 (16.7–18.9)	17.7 (16.4–18.8)	0.634	18.1 (17.2–19.0)	17.6 (16.6–18.5)	0.477	16.9 (16.7–18.6)	17.6 (16.3–18.8)	0.748
RDW birth-D7	0.9 (0.6–2.3)	0.8 (0.2–1.5)	0.289	1.5 (0.6–5.3)	1.0 (0.3–2.6)	0.217	0.7 (0.2–1.0)	0.8 (0.1–1.5)	0.798

**Table 4:** Multivariable association between RDW and BPD.

	*Univariate*			multivariable^∗^		
	OR	95% CI	*P* value	OR	95% CI	*P* value
BPD						
RDW at birth	1.145	0.851–1.541	.37	0.964	0.683–1.361	.831
RDW at D7	1.165	0.964–1.407	.113	1.054	0.835–1.332	.658
RDW at D28	1.153	0.905–1.469	.248	1.031	0.791–1.344	.822
RDW birth-D7	1.187	0.931–1.514	.167	1.119	0.829–1.511	.462
Moderate /severe BPD						
RDW at birth	1.093	0.788–1.515	.595	0.868	0.565–1.333	.518
RDW at D7	1.188	0.989–1.426	.065	0.88	0.683–1.134	.323
RDW at D28	1.059	0.790–1.420	.702	0.937	0.629–1.394	.747
RDW birth-D7	1.261	1.002–1.587	.048	0.854	0.591–1.235	.402

## 4 Discussion

The present study aimed to determine the changes in RDW values in preterm infants born at <30 weeks’ gestation and the relationship between RDW and BPD. The RDW values of infants born at <28 weeks’ GA were lower than those of infants born at 28 to 29 weeks’ GA. The RDW values in both groups increased during the first week of life, reached similar values, and remained similar throughout the first month of life. The RDW values in a month of life did not differ significantly between BPD groups.

The RDW levels of newborns are higher than those of children or adults due to active erythropoiesis and physiologic reticulocytosis.^[29,30]^ However, it is challenging to determine the normal range of RDW in preterm infants because they are changing physiologically during the perinatal period and are influenced by various conditions such as GA. The upper reference was higher in preterm infants born at less than 34 weeks’ GA than in neonates of later GA.^[29]^ The 2 previous studies reported that mean RDW was the highest at 32 to 34 weeks’ gestation and suggested that it was secondary to active erythropoiesis in the third trimester.^[30,31]^ In the present study of preterm infants with a lower GA than in the previous studies, the RDW levels at birth were higher in infants born at 28 to29 weeks’ GA than in infants born at <28 weeks’ GA. Alur et al reported similar results in that the RDW in the 26 to 31 weeks’ GA group was higher than that in the <26 weeks’ GA group, although the difference was not statistically significant.^[32]^ We suggested that RDW increased gradually, peaked at 32 to 34 weeks’ GA, and decreased again.

The RDW values in both the <28 weeks’ and ≥28 weeks’ GA groups increased during the first week of life, while the gap in RDW levels between the groups decreased. Christensen et al explained that the increase in RDW in the early days of life in preterm infants was secondary to previous RBC transfusion.^[29]^ However, the RDW showed similar trends in the present study, although the infants who received RBC transfusions in the first week of life were excluded. We cautiously speculate that preterm infants are vulnerable to postnatal environments such as inflammation and abrupt cessation of iron transplacental transportation, which introduce ineffective erythropoiesis. Christensen et al suggested that high RDW levels is due to reticulocytosis.^[29]^

In adult studies, a number of studies have reported the role of RDW as an indicator of severity or predictors of outcome for various diseases including sepsis, respiratory disease, cardiovascular disease, and critical illness.^[6,8,9,12–17,33–36]^ Although the mechanism of RDW increase has not been fully determined, it has been suggested that chronic hypoxia, malnutrition, and inflammation cause an increase in RDW values.^[4,5,37]^ Hypoxia induces erythropoietin release, which leads to the release of immature reticulocytes into the circulation.^[5]^ Injured RBCs by inflammation aggravate disease progression by decreasing oxygen transfer to organs and tissues.^[4]^

RDW values were higher in patients with chronic obstructive pulmonary disease than in healthy people^[34]^ and associated with its severity and outcomes, including mortality and readmission rates.^[8,33,34,36]^ The pathophysiology of BPD and chronic obstructive pulmonary disease are similar.^[38]^ Both result from the impairment of alveolarization/vascularization after inflammation and oxidative stress. Recently, Go et al reported that RDW at D28 can predict BPD and its severity.^[26]^ But they found RDW at birth and at D14 were not related to BPD. Although they performed multivariate logistic regression with RBC transfusion, they did not account for transfusion between D14-D28. RBC transfusion is known to affect RDW levels and can limit its role in the prediction of BPD.^[39,40]^ In addition, infants with BPD need oxygen or positive pressure ventilation and are likely to get a transfusion. As a result, including infants with transfusion may introduce a confounding factor in the analysis, and this may affect the results. Garofoli et al also showed that the RDW at the 4th week of life was higher in the BPD group than in the nonBPD group, whereas there was no difference in RDW within the first 3 days of life between the groups.^[25]^ However, they did not consider GA. In contrast, Doğan found that RDW at birth was related to BPD, which contradicts the findings of the other 2 studies.^[27]^ However, their findings were strongly different from previous studies, with high RDW levels being associated with an odds ratio of 11.9. In the present study, the RDW values at birth, D7, D28, and the change between birth to D7 were not related to the development of BPD. There was no difference even when we evaluated the data further by dividing it into 2 groups of GA, considering the difference in RDW levels and incidence of BPD in accordance to GA.

There were some limitations to this study. First, morbidities such as necrotizing enterocolitis and patent ductus arteriosus were not evaluated due to their complex relationships to BPD over time. However, we investigated whether RDW could be a predictor of BPD independently of other morbidities in a clinical setting. Second, the babies who received RBC transfusion between at birth and D7 were excluded in order to prevent them from affecting RDW values. However, this may have induced a selection bias in which more vulnerable babies were excluded. Further, while we excluded infants who got transfusions in early days of life, we still have this limitation regarding RDW at D28.

The strength of this study was in evaluating changes of RDW in early days of life as well as RDW levels at birth and D7, and their relationships with BPD presentation, even though our study did not show any statistical significance in our results. We also divided our patients into 2 groups born <28 weeks’ GA and ≥28 weeks’ GA, because both RDW levels and incidence of BPD can be quite different according to GA.

In conclusion, RDW values vary in accordance with GA and they change during the newborn period. RDW values at birth were higher in infants born at 28 to 29 weeks’ GA than in those born at <28 weeks’ GA. RDW values of both groups increased in the first week of life and remained similar during the first month of life. More caution is necessary when assessing RDW values in relation to neonatal morbidity. The RDW values in the first month of life were not associated with BPD development independent of severity in this study. Thus, the usefulness of RDW as a predictor of BPD remains unknown and requires further large-scale studies.

## Author contributions

**Conceptualization:** Seong Hee Oh, Chan-Hoo Park.

**Data curation:** Seong Hee Oh, Hyun-Jeong Do, Jae Young Cho.

**Formal analysis:** Seong Hee Oh, Hyun-Jeong Do, Jae Young Cho.

**Investigation:** Hyun-Jeong Do, Ji Sook Park, Jae Young Cho.

**Methodology:** Seong Hee Oh, Chan-Hoo Park.

**Visualization:** Seong Hee Oh, Ji Sook Park.

**Writing – original draft:** Seong Hee Oh.

**Writing – review & editing:** Seong Hee Oh, Hyun-Jeong Do, Ji Sook Park, Jae Young Cho, Chan-Hoo Park.
